# Efficacy of Certain Monoterpenes as Antifungal Agents and Abiotic Elicitors Against Chocolate Spot Disease of Faba Beans Caused by *Botrytis fabae*

**DOI:** 10.3390/pathogens15050484

**Published:** 2026-04-30

**Authors:** Aly Derbalah, Ahmed Mohamed, Nehad El-Gammal, Warda Hussain, Amany Hamza, Ahmed Alhusays, Ayman Omar, Saleh Alhewairini

**Affiliations:** 1 Department of Plant Protection, College of Agriculture and Food, Qassim University, Buraydah 51452, Saudi Arabia; ay.alhusays@qu.edu.sa (A.A.); a.mohmed@qu.edu.sa (A.O.); 2 Pesticides Chemistry and Toxicology Department, Faculty of Agriculture, Kafrelsheikh University, Kafrelshiekh 33516, Egypt; amany_mohammed@agr.kfs.edu.eg; 3 Plant Pathology Research Institute, Agriclutural Research Centre, Giza 12619, Egypt; ahmed.gaballa@yahoo.com (A.M.); nehad_elgamal655@yahoo.com (N.E.-G.); malk62974@gmail.com (W.H.)

**Keywords:** faba bean, monoterpenes, chocolate spot, resistance induction, defense enzyme

## Abstract

Chocolate spot, caused by the ascomycete fungus *Botrytis fabae*, is a devastating foliar disease and a major constraint on the quality and yield of faba beans (*Vicia faba*). Monoterpenes, such as carvone, cineole, and linalool, are often considered natural-identical alternatives to synthetic chemicals. Therefore, this study was carried out to assess the antifungal activity of some eco-friendly control agents (carvone, cineole, and linalool) against *B. fabae*, the causative agent of chocolate spot disease in faba beans, through growth inhibition assays in vitro. Furthermore, the efficacy of the tested monoterpenes for reducing the severity of chocolate spot disease in faba beans was evaluated under field conditions. Moreover, these eco-friendly control agents activate plant defense enzymes (phenylalanine ammonia-lyase, polyphenol oxidase, and peroxidase) as a self-defense mechanism against pathogen attacks of faba bean plants were investigated. Moreover, the impact of the tested monoterpenes on growth and yield characters of faba bean was evaluated. The results indicated a significant decrease in *B. fabae* growth following a treatment with the tested compounds compared to untreated controls. In field trials, treated faba bean plants exhibited a notable reduction in disease severity. Additionally, the application of monoterpenes enhanced the activity of defense enzymes (phenylalanine ammonia-lyase, polyphenol oxidase, and peroxidase), which are integral to plant defense mechanisms. Treatments also resulted in significant improvement growth and yield characters of faba bean. These findings suggest that the tested monoterpenes could serve as a control strategy for managing *B. fabae*, offering an environmentally sustainable alternative to conventional fungicides.

## 1. Introduction

Faba bean (*Vicia faba* L.), a nutritious leguminous and cool-tolerant crop, is grown all over the world. It contains minerals such as iron, zinc, and calcium, as well as vitamins B1, B2, and C. It is also utilized for fodder and forage, with protein levels ranging from 18.5 to 37.8% [1]. Furthermore, faba bean, like other legumes, improves soil fertility by fixing nitrogen, making it an ideal rotation crop for cereals and other crops [2,3]. According to Teshome and Tagegn [3], grown faba beans are mostly used as human food in developing countries and as animal feed in developed nations because of their protein content and other compounds that can help people replace animal protein [1,4,5].

One of the main factors affecting faba bean yield is the *B. fabae* attack on plants. *B. fabae* is the source of grass disease, also called chocolate spot disease, which mostly affects the north and middle Nile Delta of Egypt. It directly impacts protein synthesis, resulting in a yield output reduction of over 50% [6,7]. One of the most significant and possibly yield-limiting foliar diseases of faba beans is chocolate spot, which is known to result in significant yield losses in the world’s main bean-producing regions [8,9]. Chocolate spot is caused by *B. fabae* and infects every portion of the broad bean plant that is above the ground. Additionally, because of the current climate, chocolate spot lowers seed output and deteriorates seed quality, which causes losses [10] in the overall amount of the total carbohydrates, nitrogen, nucleic acid, and protein content of the yielded seeds [11].

Therefore, controlling this disease is considered a source of major concern. Chocolate spot is most controlled by the application of synthetic fungicides. However, due to the high costs of fungicides, detrimental impacts on the environment and human health, and the ability to destroy important soil microbiota, fungicide usage is not recommended [12]. Also, chemical fungicide overuse has the potential to create new resistant fungal strains [13,14,15]. Therefore, a strong demand is developing for these safe, natural, and eco-friendly solutions for organic farming and future crop protection strategies. Thus, research on the potential use of secondary metabolites from plants to regulate disease has received a lot of attention [16,17,18]. Pest management is a well-established application of phytochemicals, a class of compounds that include terpenoids, phenols, alkaloids, and glucosides [18,19,20,21]. Essential oils (EOs) rich in monoterpenes (MT) are the least toxic and best option for controlling diseases and pests [18,19,20,22]. Monoterpenes, which have low vapor pressure, minimal animal toxicity, and lipophilicity, possess a wide spectrum of biological actions against plant pathogens, nematodes, and weeds, according to various research [18,19,20,21,22,23,24,25]. Among monoterpenes, carvone, linalool, and cineole are known as strong antifungal compounds and are effective for controlling several plant pathogens [18,20,21].

Disease control agents can work by helping plants to develop resistance to the pathogen or by their direct antimicrobial activity. In plants, disease resistance is linked to the initiation of a broad range of defense mechanisms that reduce infection at specific phases of the host–pathogen relationship. One key defense strategy, particularly prominent in plants, involves hydrolytic enzymes such as polyphenol oxidase (PPO) and phenylalanine ammonia-lyase that are activated or synthesized upon microbial infection to produce defense responses [26,27,28]. Changes in cell metabolism, particularly the activity of defense enzymes, are brought about by the interaction between the pathogen and the host plant. The nuclear-encoded enzyme polyphenol oxidase (PPO) catalyzes the oxidation of phenols to quinones in an oxygen-dependent manner, while phenylalanine ammonia-lyase (PAL) is the essential enzyme for the synthesis of a number of secondary compounds linked to defense, including phenols and lignin [26,27,28]. EO components, such as monoterpenes, can be used in various ways to move from pathogen suppression to plant protection. One such strategy is acting as innate immunity effector molecules, which offer a potent first line of defense against infectious pathogens [29].

Therefore, the purpose of this study was to assess the antifungal activity of certain plant-derived compounds (carvone, cineole, and linalool) against *B. fabae* in a laboratory setting; determine the degree to which these compounds could suppress chocolate spot disease in faba bean plants in a field setting; find out the induction of defense responses of faba bean plants against the pathogen attack through activation of defense enzymes; and finally investigate the impact of tested control agents on certain growth and yield characteristics of faba bean.

## 2. Materials and Methods

### 2.1. Chemicals

Sigma Aldrich, Burlington, VT, USA, supplied the tested monoterpenes (carvone, cineole, and linalool) with 99% purity. Mancozeb, also known as Rich 80% WP, was produced by Kanza Group for Pesticides and Chemicals Company, Cairo, Egypt and was suggested as a fungicide for chocolate spot management in faba beans.

### 2.2. Isolate Source and Pathogenicity

A representative sample of 20–30 naturally infected faba bean (var. Giza 3) leaves (showing symptoms of chocolate spot) were gathered from fields in March of 2022 at Gemmeiza Agricultural Research Station, Gharbia governorate, Egypt. Infected leaves with chocolate spot disease symptoms were collected, cut into small pieces, and surface-sterilized for 2 min with a 3% sodium hypochlorite solution. They were cleaned many times with sterilized distilled water before drying between two layers of sterilized filter paper to eliminate excess distilled water. Pieces with a single lesion of the relevant disease were plated on faba bean leaf extract agar medium “FBLA” (extract of faba bean leaves, 30 g sucrose, 20 g sodium chloride, and 20 g agar in one liter of distilled water) as described by Hanouike and Hasanain [30]. Four pieces were placed in each Petri dish and incubated at 20 °C for 12 days until full growth in the Petri dish. The isolation process was repeated three times for confirmation. The identification was based on the cultural characteristics (i.e., colony color and formation of sclerotia on the potato dextrose agar (PDA)). The fungal isolate was confirmedly identified in the Fungal Taxonomy Research Department, Plant Pathology Research Institute, Agricultural Research Center, and had been given the name *B. fabae* EG-32. The purified cultures were preserved at 4 °C using PDA slants and used for pathogenicity test according to the methods described by Abdel-Aleem et al. [31]. The tested isolate caused the common symptom of chocolate spot disease.

### 2.3. The Antifungal Activity of the Tested Monoterpenes Against B. fabae Fungus Under In Vitro

In vitro, *B. fabae* was the target of the antifungal activity of the three monoterpenes that were tested. The percentage of inhibition in the growth of the fungal pathogen in Petri dishes, induced by the selected MT, compared to the control (untreated *B. fabae*) was used to test their efficacy. For every treatment, four concentrations of the selected MTs and mancozeb were employed. Sixty-ml sections of autoclaved PDA that had been cooled to roughly 45 °C were mixed with the appropriate stock solution to achieve the necessary concentrations (20, 40, 60, and 80 mg active ingredient (ai)/L) for the tested compounds. Due to their hydrophobicity, the tested monoterpenes were dissolved into dimethyl sulfoxide (DMSO) before mixing with PDA. The tested concentrations for mancozeb were 4, 6, 8, and 10 mg ai/L. Nine-centimeter Petri dishes were utilized in triplicate for every concentration of every treatment, including the control. The tested components were not added during the control treatment. A disk (5 mm in diameter) containing the mycelium growth from a 5-day-old *B. fabae* culture was used to inoculate the center of each dish. Parafilm was used to seal the dishes in order to prevent volatile chemicals from evaporating. The plates were incubated at 22 °C until the control plates reached full mycelial growth. Using Vincent’s proposed formula [32], the inhibition % of *B. fabae*’s radial growth was determined. Three replications of the experiment were conducted, using all concentrations for each treatment. Equation (1) illustrates the calculation of the inhibition percentage.

(1) I% = A−B/A × 100

where A = the radial growth of *B. fabae* on PDA as control and B = the radial growth of the same pathogen growing on PDA supplemented with MTs at different concentrations.

### 2.4. Field Experiments

During the two seasons (2022–2023 and 2023–2024), this experiment was carried out at the Farm of Gemmeiza Agricultural Research Station, Plant Pathology Research Institute, Agricultural Research Center, Giza, Egypt. Giza 3 was the used cultivar of faba bean, which was obtained from the Agricultural Research Center, Central Administration of Seeds. Four repetitions of the randomized complete block design were employed for this experiment. Forty-five days after sowing (beginning of January), the upper side of the leaves was inoculated with 1.5 mL of spore suspension containing about 5 × 10^5^ spores per ml of *B. fabae* EG-32, one droplet on each half of the leaflet using a micropipette, amended with Tween 20^®^ (1.2% *v*/*v*) and covered with polyethylene bags for 24 h to maintain high relative humidity [33]. Plants sprayed with distilled water were used as a control. In both seasons, fungicide at 2 g ai/L and the three treatments (carvone, linalool, and cineole) at 0.060 and 0.080 g ai/L were applied three times between mid-January and the end of February. Water-sprayed leaves served only as a control. Depending on the ideal spraying conditions, the first, second, and third application intervals varied from 15 to 17 days. The meteorological data, including temperatures (°C), relative humidity (%), wind speed (m/s), and rainfall (mm/day) during the two seasons of study, were recorded daily and their monthly mean values are presented in Table 1.

A disease assessment was conducted one week after the last spray, as described by Hanouike and Hasanain’s [30] approach to assess the disease severity of the chocolate spot on faba beans. The plants from each plot were subjected to a severity rating system ranging from 0 to 9. A score of 0 indicates no visible leaf infection, while a score of 8 indicates disease covering less than 10%, 20%, 30%, 40%, 50%, 60%, 70%, or 80% of the foliar tissue, and a score of 9 indicates disease covering more than 80% of the foliar tissue.

Disease Severity % = ∑(n × v)/9 N × 100

where n = number of plants in each severity class, v = numerical grade (disease severity class), N = total number of plants, and 9 = maximum disease severity class.

Finally, the reduction in disease severity (efficacy%) was calculated using the following Equation:

Efficacy % = C−T/C × 100

where C = disease severity % in the control and T = disease severity % in the treatment.

After 120 days of sowing, 10 plants were harvested by hand, and the fresh weight was measured. After that, the harvested plants were allowed to dry for 5 days under natural conditions before being measured for plant height (cm), root length, dry weight, number of pods per plant, weight of 100 grains (g), and biological yield [kg/plot] using 10 plants per plot.

### 2.5. Biochemical Analysis

#### 2.5.1. Determination of Chlorophyll

Each leaf sample consisted of five leaf disks with a diameter of one centimeter (three plants from each replicate of each treatment and five leaves from each plant) that were extracted using five milliliters of N,N dimethylformamide. The leaves were then left at room temperature for 48 h in the dark before being measured spectrophotometrically [34]. Using a spectrophotometer, the absorbance of chlorophyll A and B was measured at 645 and 663 nm, respectively. The following formulas were used to determine the amounts of chlorophyll A and B.

Ch.a = 12.64(A663)−2.49(A645)

Ch.b = 5.6(A663) − 23.26(A645)

#### 2.5.2. Sample Preparation and Extraction for Determination of the Enzyme’s Activities

After three days of the first spray, 0.5 g of faba bean leaves (three plants from each replicate and five leaves from each plant) were harvested for each treatment and kept at −80 °C until needed. Every step of the enzyme extraction process was carried out at 4 °C. Using a mortar and 0.1 M sodium phosphate buffer at a pH of 7.1 (2 mL buffer/g tissue), the sample was ground. After straining these triturated tissues through four layers of cheesecloth, the filtrates were centrifuged for 20 min at 6 °C at 3000 rpm. A UV-vis spectrophotometer (Shimadzu 3700 Model, Shimadzu Limited Company, Tokyo, Japan) was used to perform enzyme assays for polyphenol oxidase, peroxidase, and phenylalanine ammonia-lyase on the supernatant fluid. With the exception of the enzyme extract, every component in the reference cuvette was present at the same concentration as in the sample cuvette. After fully mixing the spectrophotometer cuvettes, the measurements were taken every 30 s for two minutes. An enzyme’s activity was measured in terms of change in absorbance per gram per minute.

#### 2.5.3. Polyphenol Oxidase Assay

The polyphenol oxidase (PPO) activity was established following the method of Galeazzi et al. [35]. Using this procedure, an ultraviolet spectrophotometer was used to detect the absorbance at 398 nm after 100 μL of enzyme extract was incubated for 2 min at 24 °C with 2 mL of 0.05 M phosphate buffer (pH 7.0) and 0.5 mL of 0.5 M catechol. The PPO activity was reported as U398 = 0.01ΔOD398, where ΔOD is the change in optical density per milligram of protein per minute and U398 is the enzyme unit at 398 nm. Protein content was determined using a nanodrop ND-1000 spectrophotometer at OD280.

#### 2.5.4. Peroxidase Assay

The activity of peroxidase (POD) was measured with guaiacol acting as a substrate. A total of 0.1 mL of crude extract and 2 mL of guaiacol (8 mM) in 100 mM sodium phosphate buffer (pH 6.4) made up the reaction mixture, which was incubated for 30 min at 30 °C. Following the addition of 1 milliliter of H_2_O_2_ (24 mM), the increase in absorbance at 460 nm was recorded. According to Ippolito et al. [36], the POD activities were expressed as U460, where U460 = 0.01ΔOD460, and where ΔOD is the change in optical density per minute and U460 is the enzyme unit at 460 nm. Protein content was determined using a nanodrop ND-1000 spectrophotometer at OD280.

#### 2.5.5. Phenylalanine Ammonia-Lyase (PAL) Assay

The faba bean leaf acetone powder (0.12 g) was used to prepare the enzymes, which were then mixed for a minute in 100 milliliters of acetone. After passing the homogenate through Whatman No. 1 filter paper, acetone was used to re-blend the residue. This process was carried out three times. After three hours of air drying at room temperature, the acetone powder was kept at −18 °C until needed. A 500 mg of dry acetone powder was added to 10 mL of cold 0.1 µM borate buffer (pH 8.8) for the PAL experiment, and the mixture was agitated for one hour at 4 °C. The suspension was dialyzed for 48 h at 4 °C in 0.2 µ borate buffers at pH 8.8 after being centrifuged twice at 6000 rpm at 4 °C according to the method described by Lisker et al. [37]. The PAL activity was determined as phenol components in µmol/min/g fresh weight.

### 2.6. Statistical Analysis

To evaluate the relation between the independent variable (treatments) and dependent variable (growth inhibition), a regression analysis was employed using SPSS software version 15. Field data were analyzed statistically through the one-way ANOVA test with a *p* value of ≥0.05 using SPSS software version 15. After the ANOVA test, the different means were compared using Tukey range tests. The data of each season were analyzed separately.

## 3. Results

### 3.1. Antifungal Activity of the Tested Monoterpenes Against B. fabae In Vitro

**Figure 1:**
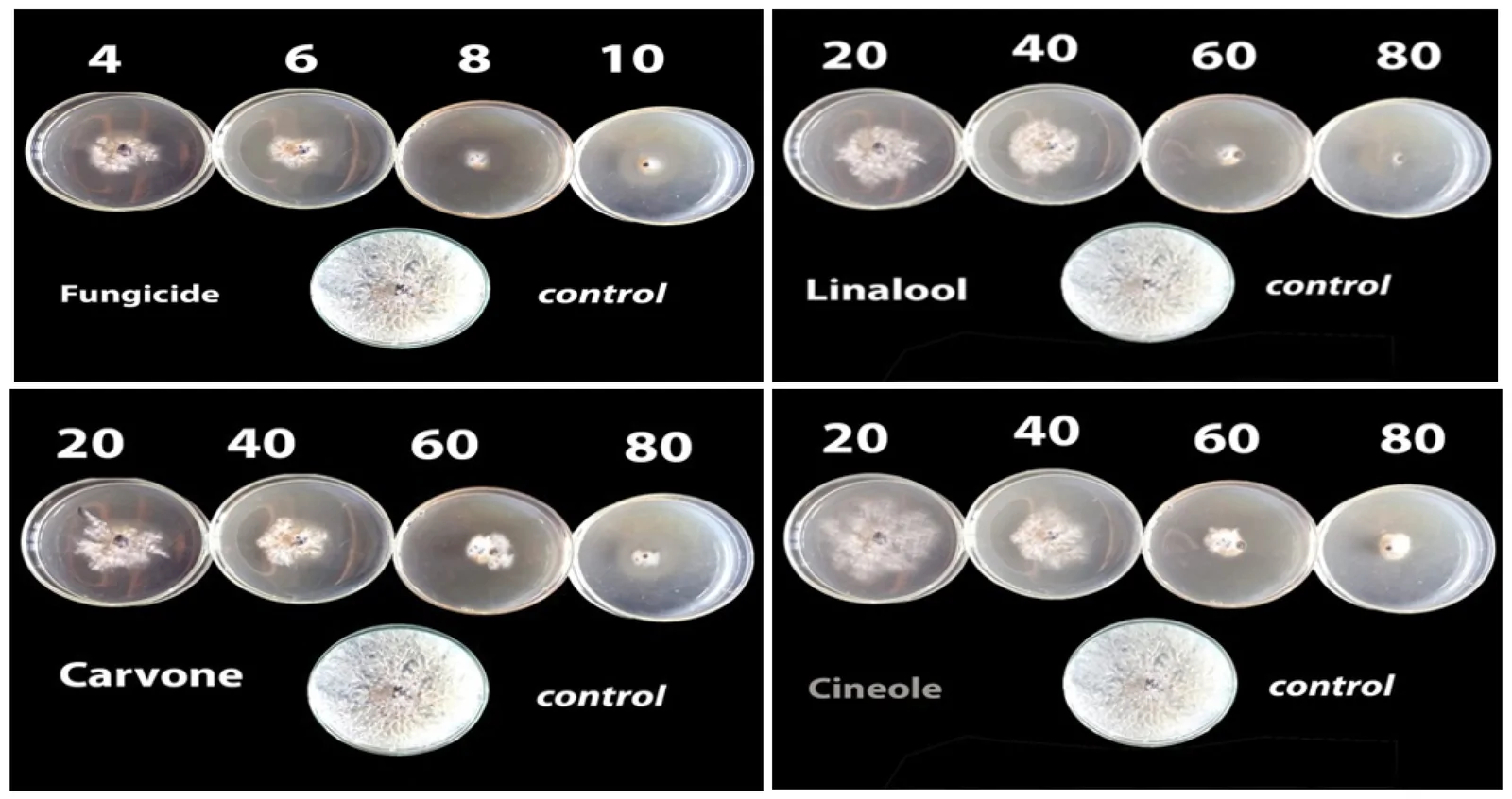
Radial growth of *B. fabae* colony in Petri dishes containing 2% PDA melted with cineole, carvone, linalool, and mancozeb at different concentrations, compared to the control (PDA only).

Table 2 and Figure 1 show the effect of the tested monoterpenes on the mycelial development of *B. fabae* compared to the control. The results demonstrated that the tested monoterpenes greatly suppressed *B. fabae* growth as compared to the untreated control. Linalool proved to be the most effective treatment, followed by carvone and cineole. Furthermore, the inhibition percentages in fungus growth treated with the investigated compounds correlated strongly with their concentrations.

### 3.2. Efficacy of the Tested Control Agents Against Chocolate Spot in Faba Bean Under Field Conditions

Table 3 shows the efficacy of the tested monoterpenes versus the recommended fungicide in terms of disease severity reduction compared to the control. The results revealed that the recommended fungicide was the most effective treatment against chocolate spot, followed by linalool, carvone, and cineole in both growing seasons (2022–2023 and 2023–2024). The effectiveness of the tested control agents increased with concentration. Furthermore, the tested control agents performed better in the second season than in the first season.

### 3.3. Effect of the Tested Treatments on Chlorophyll Content of Faba Bean

Table 4 indicates the effect of the tested monoterpenes and chemical fungicide on chlorophyll content (chlorophyll A, chlorophyll B, and chlorophyll A and B) of faba bean plants during the two tested seasons (2022–2023 and 2023–2024) under field conditions. In both growth seasons, the tested treatments significantly increased the chlorophyll content of the faba bean plants compared to the untreated control (*p* = 0.05). In both growth seasons, fungicide was shown to have the maximum chlorophyll content in treated faba bean plants, followed by linalool, cineole, and carvone. The concentration at which the tested monoterpenes were applied increased in parallel with a steady increase in chlorophyll content during both growth seasons. The measured chlorophyll content was higher in the second season than the first under all investigated treatments.

### 3.4. Effect of the Tested Treatments on Enzyme Activity of Faba Bean

Table 5 shows the influence of the tested monoterpenes and chemical fungicide on the activity of certain enzymes (peroxidase, polyphenol oxidase, and phenylalanine ammonia-lyase) during the two tested seasons (2022–2023 and 2023–2024) in field settings. In both growing seasons, the tested treatments considerably increased (*p* = 0.05) the measured enzyme activity of faba bean when compared to the control treatment. Linalool had the highest activity of the tested enzymes in faba bean, followed by fungicide, carvone, and cineole in both seasons. In both growing seasons, there was a progressive increase in enzyme activity that corresponded to an increase in the rate of monoterpene concentration. The observed enzyme activity was higher in the second season compared to the first season under all investigated treatments.

### 3.5. Effect of the Tested Monoterpenes on Some Growth Parameters of Faba Bean

Table 6 illustrates the influence of the tested monoterpenes and chemical fungicide on several growth parameters (mean plant height, root shoot length, and fresh and dry weight) throughout two seasons (2022–2023 and 2023–2024) in the field. In both growing seasons, the treatments tested considerably (*p* = 0.05) improved faba bean growth characteristics over the control treatment. Linalool produced the best yield characteristics in faba bean, followed by carvone, fungicide, and cineole, in both growth seasons. Increasing the rate of application of the tested monoterpenes resulted in a progressive increase in every parameter across both growth seasons. The assessed growth characteristics were higher in the second season compared to the first season under all investigated treatments.

### 3.6. Effect of the Tested Treatments on Some Yield Parameters of Faba Bean

Table 7 shows the impact of the chemical fungicide and tested monoterpenes on several faba bean yield parameters during the course of the two testing seasons (2022–2023 and 2023–2024) in the field. The impact of treatments on the mean number of pods per plant, the mean weight of 100 grains (g), and the mean weight of grains per plot were displayed in Table 7. Compared to the control, the investigated treatments considerably (*p* = 0.05) improved the faba bean yield characteristics during both growth seasons. In both growth seasons, linalool produced the best yield characteristics for faba beans, followed by fungicide, carvone, and cineole, in that order. As the rate of application increased, a progressive increase in all parameters was seen in both growing seasons. Under all investigated treatments, the measured yield characteristics were higher in the second season than in the first.

## 4. Discussion

Currently in use, synthetic chemical fungicides are risky for public health. Therefore, a great deal of research has been done to assess the effectiveness of biocontrol agents and plant secondary metabolites, such as essential oils and other volatile aromatic products, against plant pathogenic fungi [18,21,24,25]. This investigation assessed the antifungal efficacy of some plant-origin compounds (carvone, cineole, and linalool) against *B. fabae.* According to the findings, the examined monoterpenes exhibited fungicidal activity in a lab settings and inhibited the growth of *B. fabae* at various concentrations. This is consistent with certain research findings that showed the antifungal properties of various monoterpenes, including camphor, carvone, 1,8-cineole, fenchone, geraniol, linalool, and menthol, against fungi, including *Rhizoctonia solani*, *B. cinerea*, and *Fusarium oxysporum*, [18,21,24]. Furthermore, Tegegn et al. [38] found that *Schinus molle* extracts containing monoterpenes have a negative impact on *B. fabae* and significantly reduce mycelial growth when compared to the control under lab conditions. Ibrahim and Al-Naser [39] found that *S. molle* fruit extracts inhibited the growth of *B. cinerea* and contained terpenoids, including α-pinene, β-pinene, α-phellandrene, β-phellandrene, and limonene. Monoterpenes, or EOs, have recently been described as having various mechanisms of action on fungal species, such as ruptured cell walls and membrane disruption, inhibition of chitin synthesis, ROS accumulation, mitochondrial dysfunction, and inhibition of some specific enzyme activities [21,40,41,42].

Our field research revealed that faba bean plants treated with mancozeb and the tested monoterpenes had significantly lower disease severity when compared to the untreated control. This validates the findings of El-Nagar et al. [43], who demonstrated that the application of plant extracts containing monoterpenes significantly reduced the severity of *B. fabae* in faba beans. Additionally, Tegegn et al. [38] found that the sprayed faba bean infected with *B. fabae* with *S. molle* extracts and the positive control synthetic fungicide, mancozeb, significantly reduced the severity of *B. fabae*. Our field results revealed slight variation in the efficacy of the tested control agents between both growing seasons, and this may be due to the fact that the efficacy varies significantly season to season due to changing weather (rain, humidity, and temperature), pathogen pressure (disease intensity), and host plant factors (growth stage and variety) [44].

In this study, to analyze the pathways involved in monoterpene-induced resistance in faba bean, the activity of three defense-related enzymes was measured in treated faba beans. In contrast to the control group, the application of monoterpenes significantly increased defense-related enzymes in this faba bean study. A number of enzymes, such as POD, PAL, and PPO, are essential for induced plant defense against biotic and abiotic stressors [18,27,28,45]. Therefore, the mechanisms of activating resistance in plants include increased peroxidase activity, β-1,3-glucanase, chitinase, phenylalanine ammonia-lyase, and polyphenol oxidase, leading to systemic acquired resistance (SAR) and induced systemic resistance (ISR) [46]. Phenylalanine ammonia-lyase (PAL) enzyme is an essential enzyme in secondary metabolites of plant biosynthesis, such as the production of many phenolic compounds (coumarin, lignin, flavonoids, phenolic acids, and tannins) that lead to an increase in lignin deposition and the thickness of cell walls. Entering the second pathway, phenylpropanoid, which converts phenylalanine into trans-cinnamic acid, and the latter is converted into many phenolic compounds necessary for physiological processes, plant growth and development, and interactions between the environment and plants, especially resistance to pathogens such as lignin precipitation and structural strengthening [47,48,49]. These findings show that plant-origin monoterpenes may act as inducers of the defense-related enzymes in plants when co-applied with pathogens, especially at low concentrations, which are considered to be low in phytotoxicity, priming or elicitating defense mechanisms [27,28,50,51,52]. According to these results, monoterpenes may act as a resistance elicitor in faba bean plants to prevent the attack of the chocolate spot pathogen.

Chocolate spot disease, which drastically reduces seed yield, is the main faba bean plant disease affecting Egypt, according to Sahar et al. [53]. The results of our study showed that the application of the studied monoterpenes improved the growth and yield characteristics of faba bean plants when compared to untreated control. This is in line with the results of Abdel-Mawgoud et al. [54], who found that the numerous growth indices of watermelon plants were improved through the application of plant extract containing phenolic compounds, such as monoterpenes. Shehata et al. [55] researching celeriac plants, Fawzy et al. [56] researching Chinese garlic plants, and Hernández et al. [57] researching tomatoes discovered in the same context that applying botanical extracts containing phenolic compounds, like the monoterpenes that are under investigation in this study, as a foliar spray gave the highest values for vegetative growth. Similar to this, biostimulants with essential oils, like the monoterpenes used in this study, may increase the productivity of numerous plants by improving their ability to absorb minerals and nutrients and to grow vegetatively [18,58,59]. It was found that the monoterpenes under investigation had favorable effects on faba bean production and growth parameters. Therefore, using eco-friendly monoterpenes can be regarded as a good production strategy to get high yields of nutrient-dense vegetables with minimal environmental impact [60].

This work describes a novel method of managing *B. fabae* during the faba bean production process by using specific bioactive monoterpenes. This technology works best in production systems with high safety and low persistence as a residue because the majority of constituents in essential oils have these characteristics. The ongoing advancement of this technology will benefit sustainable or organic cropping systems in which the use of chemical pesticides for control is not preferred [61]. For the purpose of controlling plant pathogenic fungi, post-harvest fungi, and food preservation, EOs are marketed as fungicide substitutes [18,21,62,63]. In this context, it is crucial to look for safe, non-toxic control agents with a high LD_50_ on mammals that can be used in small amounts to decrease the economic losses caused by these plant pathogens. An additional consideration concerns their persistence and application methods are limited due to their low molecular weight, hydrophobicity, and high volatility. To overcome these limitations, much work is needed regarding formulation techniques to allow the release of a control profile. A recent promising domain is the formulation of nanoemulsion using bio-based surfactants [64], as well as other encapsulation techniques [65].

## 5. Conclusions

Under laboratory conditions, a treatment with the tested compounds significantly inhibited the growth of *B. fabae* in comparison to the untreated control. In field settings, faba bean plants treated with the tested compounds showed a significant reduction in *B. fabae* disease severity when compared to untreated control plants. The tested compounds improved the resistance induction of faba bean by increasing the level of self-defense enzymes. The fungicide and tested control agents greatly enhanced the faba bean’s growth and yield characteristics. The use of monoterpenes for sustainable agricultural practices appears promising, and extensive research will probably clarify or deny their relevance in diverse applications. Due to their inherent characteristics, the pest control properties are usually very transitory and less effective than synthetic products. However, monoterpenes can be an efficient alternative to conventional plant protection products when properly formulated and integrated with other pest management strategies.

## Figures and Tables

**Table 1:** Meteorological data of the field experiment in 2021/2022 and 2022/2023 growing seasons.

Month	^a^T-max(°C)		^b^T-min(°C)		^c^R.H. (%)		^d^W.S. (ms^−1^)		^e^R.F. (mm/day)	
	1st	2nd	1st	2nd	1st	2nd	1st	2nd	1st	2nd
January	18.60	29.75	8.1	8.1	71.15	69.34	6.0	5.65	0.42	0.36
February	19.34	22.44	7.18	8.41	66.22	62.45	5.56	6.10	1.01	0.91
March	31.10	30.23	11.15	10.21	58.44	59.55	6.96	6.65	0.00	0.01

Source: Water Requirement and Field Irrigation Research Department, Water and Soil Research Institute, Agricultural Research Center, Egypt. ^a^(T-max) maximum temperatures (°C), ^b^(T-min) minimum temperatures (°C), ^c^R.H.: relative humidity (%), ^d^W.S.: wind speed (m/s), ^e^R.F.: rainfall (mm/day).

**Table 2:** Radial growth and inhibition percentage of the tested monoterpenes and fungicide against *B. fabae* in vitro with regression equation and degree of correlation.

Treatments	Conc. (mg ai/L)	Inhibition%	Regression Equation	R^2^
Fungicide	4	55 ± 1.34 ^cd^	y = 7.7x + 24.1	0.99
	6	69 ± 2.21 ^ef^		
	8	88 ± 3.12 ^f^		
	10	100 ± 0.0 ^h^		
Linalool	20	55.12 ± 1.30 ^cd^	y = 0.785x + 38.5	0.98
	40	67.66 ± 2.10 ^e^		
	60	89.66 ± 0.76 ^f^		
	80	100.00 ± 0.00 ^f^		
Cineole	20	32.21 ± 1.76 ^a^	y = 0.995x + 12	0.99
	40	50.44 ± 2.22 ^bc^		
	60	75.88 ± 1.98 ^f^		
	80	90.77 ± 1.22 ^f^		
Carvone	20	48.21 ± 1.52 ^b^	y = 0.905x + 28	0.98
	40	60.77 ± 2.42 ^d^		
	60	85.33 ± 3.12 ^g^		
	80	100 ± 0.0 ^h^		
Control	0.0	0.00 ^i^	-	-

Each mean value came from three replicates followed the standard error (±SE). Different letters represent significant differences obtained using the Tukey test at *p* ≤ 0.05.

**Table 3:** Efficacy of the tested monoterpenes and fungicide against chocolate spot disease in two growing seasons.

Treatment	Conc. (g ai/L)	Season 1		Season 2	
		Disease Severity	Reduction	Disease Severity	Reduction
Linalool	0.060	20.36 ± 1.65 ^bcd^	46.08 ± 1.34	18.96 ± 2.48 ^cde^	46.77 ± 2.11
	0.080	16.56 ± 2.09 ^b^	56.14 ± 2.11	14.46 ± 1.64 ^b^	59.40 ± 3.14
Cineole	0.060	22.46 ± 1.49 ^d^	40.51 ± 1.27	21.44 ± 1.98 ^e^	39.80 ± 2.23
	0.080	18.65 ± 1.81 ^bcd^	50.60 ± 3.10	17.38 ± 1.2 ^bcd^	51.20 ± 1.34
Carvone	0.060	21.36 ± 0.98 ^cd^	43.43 ± 1.76	20.56 ± 1.91 ^de^	42.27 ± 1.65
	0.080	18.05 ± 0.98 ^bc^	52.19 ± 1.25	16.45 ± 1.83 ^bc^	53.18 ± 1.34
Fungicide	2.0	9.35 ± 1.17 ^a^	75.4 ± 1.54	7.54 ± 1.36 ^a^	78.83 ± 2.45
Control	0.00	37.76 ± 3.81 ^f^	75.23 ± 2.45	35.62 ± 2.01 ^f^	0.00

Values shown are the means and standard errors (±SE) of four replicates. Statistical comparisons were made among treatments within a single column. Different letters represent significant differences obtained using the Tukey test at *p* ≤ 0.05.

**Table 4:** Effect of the tested monoterpenes and fungicide on chlorophyll content in faba bean in two growing seasons.

Treatments	Concentration (mg/L)	Season 1		Season 2	
		Chlorophyll A	Chlorophyll B	Chlorophyll A	Chlorophyll B
Linalool	0.060	2.40 ± 0.26 ^abc^	5.52 ± 0.03 ^bcd^	2.43 ± 0.02 ^bc^	5.55 ± 0.13 ^b^
	0.080	2.49 ± 0.08 ^c^	5.76 ± 0.28 ^d^	2.51 ± 0.02 ^cd^	5.80 ± 0.1 ^cd^
Cineole	0.060	2.20 ± 0.1 ^a^	5.12 ± 0.05 ^a^	2.25 ± 0.05 ^a^	5.17 ± 0.12 ^a^
	0.080	2.44 ± 0.01 ^bc^	5.61 ± 0.27 ^cd^	2.47 ± 0.05 ^cd^	5.62 ± 0.03 ^bc^
Carvone	0.060	2.25 ± 0.1 ^ab^	5.17 ± 0.17 ^ab^	2.28 ± 0.05 ^a^	5.22 ± 0.07 ^a^
	0.080	2.43 ± 0.03 ^bc^	5.53 ± 0.09 ^bcd^	2.47 ± 0.08 ^cd^	5.55 ± 0.10 ^b^
Fungicide	2.0	2.53 ± 0.09 ^c^	5.87 ± 0.37 ^d^	2.56 ± 0.11 ^d^	5.92 ± 0.12 ^d^
Control	0.00	2.31 ± 0.03 ^abc^	5.23 ± 0.06 ^abc^	2.33 ± 0.03 ^ab^	5.26 ± 0.11 ^a^

Values shown are the means and standard errors (±SE) of four replicates. Statistical comparisons were made among treatments within a single column. Different letters represent significant differences obtained using Tukey test at *p* ≤ 0.05.

**Table 5:** Effect of the tested monoterpenes and fungicide on some self-defense enzymes in faba bean in two growing seasons.

Treatments	Conc. (mg/L)	Season 1			Season 2		
		POD	PPO	PAL	POD	PPO	PAL
Linalool	0.060	0.51 ± 0.12 ^b^	0.14 ± 0.01 ^bc^	1.51 ± 0.02 ^c^	0.54 ± 0.02 ^b^	0.15 ± 0.02 ^bc^	1.48 ± 0.01 ^c^
	0.080	0.65 ± 0.02 ^d^	0.17 ± 0.01 ^e^	1.59 ± 0.02 ^c^	0.67 ± 0.02 ^d^	0.18 ± 0.02 ^c^	1.60 ± 0.01 ^e^
Cineole	0.060	0.48 ± 0.03 ^b^	0.14 ± 0.01 ^bc^	1.42 ± 0.02 ^b^	0.52 ± 0.02 ^b^	0.14 ± 0.02 ^b^	1.39 ± 0.02 ^b^
	0.080	0.57 ± 0.02 ^c^	0.14 ± 0.01 ^bc^	1.53 ± 0.02 ^c^	0.58 ± 0.02 ^c^	0.15 ± 0.01 ^bc^	1.51 ± 0.02 ^cd^
Carvone	0.060	0.51 ± 0.03 ^b^	0.13 ± 0.01 ^b^	1.39 ± 0.02 ^b^	0.53 ± 0.02 ^b^	0.15 ± 0.02 ^bc^	1.42 ± 0.01 ^b^
	0.080	0.58 ± 0.04 ^c^	0.15 ± 0.01 ^cd^	1.56 ± 0.01 ^c^	0.59 ± 0.03 ^c^	0.18 ± 0.02 ^c^	1.53 ± 0.01 ^d^
Fungicide	2.0	0.63 ± 0.03 ^d^	0. 16 ± 0.01 ^de^	1.68 ± 0.02 ^d^	0.66 ± 0.02 ^d^	0.18 ± 0.01 ^c^	1.67 ± 0.01 ^f^
Control	0.00	0.39 ± 0.008 ^a^	0.08 ± 0.001 ^a^	1. 09 ± 0.01 ^a^	0.44 ± 0.02 ^a^	0.11 ± 0.01 ^a^	1.03 ± 0.01 ^a^

Values shown are the means and standard errors (±SE) of four replicates. Statistical comparisons were made among treatments within a single column. Different letters represent significant differences obtained using the Tukey test at *p* ≤ 0.05.

**Table 6:** Effect of the tested monoterpenes and fungicide on some growth characteristics in faba bean in two growing seasons.

Treatments	Conc. (mg/L)	Season 1		Season 2	
		Plant Height cm	Root Length	Plant Height cm	Root Length
Linalool	0.060	98.12 ± 2.90 ^bc^	11.14 ± 0.04 ^c^	100.20 ± 2.15 ^b^	11.65 ± 0.1 ^bc^
	0.080	112.23 ± 2.11 ^e^	12.32 ± 0.39 ^f^	115.32 ± 2.37 ^e^	12.74 ± 1.02 ^c^
Cineole	0.060	95.16 ± 2.89 ^b^	11.42 ± 0.02 ^cd^	98.54 ± 0.97 ^b^	11.53 ± 1.04 ^bc^
	0.080	102.65 ± 3.74 ^cd^	10.75 ± 0.25 ^b^	104.34 ± 2.36 ^c^	1153 ± 0.02 ^bc^
Carvone	0.060	96.45 ± 4.38 ^b^	10.82 ± 0.02 ^b^	98.32 ± 2.68 ^b^	10.97 ± 0.44 ^ab^
	0.080	107.21 ± 2.17 ^de^	11.76 ± 0.13 ^e^	108.6 ± 1.85 ^d^	11.68 ± 0.31 ^bc^
Fungicide	2.0	105.84 ± 3.36 ^d^	11.52 ± 0.11 ^de^	107.45 ± 2.09 ^cd^	11.32 ± 0.29 ^ab^
Control	0.00	83.34 ± 2.37 ^a^	10.09 ± 0.04 ^a^	85.10 ± 1.43 ^a^	10.12 ± 0.12 ^a^

Values shown are the means and standard errors (±SE) of four replicates. Statistical comparisons were made among treatments within a single column. The different letters represent significant differences obtained using Tukey test at *p* ≤ 0.05.

**Table 7:** Effect of the tested monoterpenes and fungicide on some yield characters in faba bean in two growing seasons.

Treatments	Conc. (mg/L)	Season 1			Season 2		
		Weight of 100 Grains	Pod Number	Kg Plot	Weight of 100 Grains	Pod Number	Kg Plot
Linalool	0.060	76.74 ± 1.44 ^ab^	6.9 ± 0.20 ^bc^	39.88 ± 1.32 ^abcd^	77.61 ± 1.39 ^ab^	7.1 ± 0.1 ^bc^	39.97 ± 1.79 ^a^
	0.080	81.45 ± 0.75 ^d^	7.6 ± 0.26 ^e^	42.63 ± 1.63 ^d^	82.53 ± 2.20 ^c^	7.8 ± 0.2 ^e^	43.65 ± 1.38 ^b^
Cineole	0.060	75.64 ± 1.45 ^a^	6.7 ± 0.43 ^e^	38.15 ± 0.79 ^a^	77.23 ± 1.19 ^ab^	6.9 ± 0.2 ^b^	39.56 ± 0.96 ^a^
	0.080	77.34 ± 1.86 ^abc^	7.1 ± 0.2 ^cd^	40.65 ± 2.33 ^abcd^	78.18 ± 2.76 ^ab^	7.3 ± 0.3 ^cd^	41.54 ± 0.79 ^ab^
Carvone	0.060	76.12 ± 3.12 ^ab^	6.6 ± 0.1 ^ab^	39.17 ± 1.13 ^abc^	77.83 ± 1.15 ^ab^	6.8 ± 0.2 ^ab^	39.83 ± 0.83 ^a^
	0.080	79.38 ± 2.79 ^bcd^	7.4 ± 0.1 ^de^	41.23 ± 0.74 ^bcd^	79.91 ± 1.45 ^bc^	7.3 ± 0.2 ^cd^	41.64 ± 1.06 ^ab^
Fungicide	2.0	80.45 ± 0.76 ^cd^	7.5 ± 0.2 ^e^	41.47 ± 0.76 ^cd^	82.25 ± 2.72 ^c^	7.5 ± 0.1 ^de^	42.23 ± 0.87 ^b^
Control	0.00	75.16 ± 0.66 ^a^	6.3 ± 0.1 ^a^	38.56 ± 2.02 ^ab^	75.16 ± 1.73 ^a^	6.5 ± 0.1 ^a^	39.45 ± 1.06 ^a^

Values shown are the means and standard errors (±SE) of four replicates. Statistical comparisons were made among treatments within a single column. The different letters represent significant differences using Tukey test at *p* ≤ 0.05.

## Data Availability

The original contributions presented in this study are included in the article. Further inquiries can be directed to the corresponding authors.
